# The large‐scale removal of mammalian invasive alien species in Northern Europe

**DOI:** 10.1002/ps.4224

**Published:** 2016-02-09

**Authors:** Peter A Robertson, Tim Adriaens, Xavier Lambin, Aileen Mill, Sugoto Roy, Craig M Shuttleworth, Mike Sutton‐Croft

**Affiliations:** ^1^ Animal and Plant Health AgencySand HuttonYorkUK; ^2^ School of BiologyNewcastle UniversityNewcastleUK; ^3^ Instituut voor Natuur‐ en Bosonderzoek – Research Institute for Nature and Forest (INBO)BrusselsBelgium; ^4^ Zoology BuildingUniversity of AberdeenAberdeenUK; ^5^ IUCNGlandSwitzerland; ^6^ School of EnvironmentNatural Resources and GeographyBangor UniversityBangorUK

**Keywords:** invasive species, alien species, eradication, mammal, control

## Abstract

Numerous examples exist of successful mammalian invasive alien species (IAS) eradications from small islands (<10 km^2^), but few from more extensive areas. We review 15 large‐scale removals (mean area 2627 km^2^) from Northern Europe since 1900, including edible dormouse, muskrat, coypu, Himalayan porcupine, Pallas' and grey squirrels and American mink, each primarily based on daily checking of static traps. Objectives included true eradication or complete removal to a buffer zone, as distinct from other programmes that involved local control to limit damage or spread. Twelve eradication/removal programmes (80%) were successful. Cost increased with and was best predicted by area, while the cost per unit area decreased; the number of individual animals removed did not add significantly to the model. Doubling the area controlled reduced cost per unit area by 10%, but there was no evidence that cost effectiveness had increased through time. Compared with small islands, larger‐scale programmes followed similar patterns of effort in relation to area. However, they brought challenges when defining boundaries and consequent uncertainties around costs, the definition of their objectives, confirmation of success and different considerations for managing recolonisation. Novel technologies or increased use of volunteers may reduce costs. Rapid response to new incursions is recommended as best practice rather than large‐scale control to reduce the environmental, financial and welfare costs. © 2016 Crown copyright. *Pest Management Science* published by John Wiley & Sons Ltd on behalf of Society of Chemical Industry.

## 1 INTRODUCTION

Invasive alien species (IAS) have significant impacts on native ecosystems,^1^, ^2^ are responsible for a wide range of conflicts with human activities^3^, ^4^ and are a major cause of native species extinctions.^5^ The introduction of IAS can disturb natural communities, and although it may increase local species number, it often has wider impacts through competition for resources, habitat alteration, predation and disease transmission.

Terrestrial vertebrates have attracted particular attention as IAS. A review of the effects of IAS from different taxonomic groups on ecosystem services^6^, ^7^ concluded that terrestrial vertebrates were responsible for the greatest range of impacts. Vertebrates are also recognised as having a high potential to become invasive following introduction.^8^ They have also often been the particular focus of management efforts, including eradications.^9^, ^10^, ^11^ However, it is recognised that the regulatory and technical tools addressing eradication, control or management of invasive species remain poorly developed.^12^

The most effective way to manage the impacts caused by biological invasions is the prevention of new unwanted introductions, but once this has failed, early eradication is the best alternative, considering the costs and undesired effects related to permanent control or to a ‘do nothing’ policy.^13^, ^14^ Current efforts to manage the impacts of IAS, though effective in particular situations, are not addressing the underlying problem of continued presence,^15^ and consequently there have been calls that eradication should be more widely applied as a management tool.^16^

Most documented eradications relate to islands, with 89% of reported European eradications^9^, ^10^ taking place in these areas. It is important to understand the basic assumptions of eradication, and how this influences success.^17^ For island eradications, the costs and effectiveness are well described.^10^, ^18^, ^19^, ^20^, ^21^ However, the number of eradications or wide‐scale control programmes of mammalian IAS on larger land masses remains small, and the factors affecting their success or cost have only been described at an individual project level.

Eradication has been defined as the complete and permanent removal of all wild populations of an alien plant or animal species from a defined area by means of a time‐limited campaign.^22^ These authors also emphasise the distinction between eradication and control to keep damage at an acceptable level and containment to limit spread. This definition can be readily applied to islands, where the area can be readily defined and permanent removal is a reasonable objective. However, when dealing with IAS over larger areas, a wider range of objectives are apparent than simply eradication, containment or control. These may include maintaining cleared areas through control in neighbouring buffer zones, or ongoing surveillance and intermittent control to prevent the establishment of new populations. These management approaches are not true eradications, as control may not be limited to a defined area and time and the risk of recolonisation remains. However, they do not simply aim to keep damage at an acceptable level, or provide containment to limit spread, and hence do not fit clearly with the published description.^22^ Similarly, funding availability can lead to a sequence of projects, each with limited objectives, but which may culminate in eradication over time.

New European legislation^23^ will require member states to act if defined IAS establish populations within their territories, although the nature of the required action has yet to be clarified. This will add statutory weight to the need for control, and increase the need for effective methods to deal with such species on larger land masses as well as in island situations. This reinforces the need to gain a better understanding of the practice of wide‐scale mammalian IAS control. Outside Europe, initiatives such as Predator‐free New Zealand^24^ will also require cost‐effective control strategies over larger contiguous land areas.

Great Britain, Ireland and Belgium have a long history of controlling large‐scale mammalian IAS, starting in 1900. These programmes, conducted at a regional or national level, provide insights into the costs and effectiveness of control in larger land masses. They have also demonstrated a variety of objectives, reflecting different approaches to the challenges of control over large areas. Although described in the literature, the costs and effectiveness of these large programmes have not been presented in a consistent format. In this paper we present data on the effort and associated costs encountered, and contrast these with the published literature on smaller areas, typically islands. We discuss the challenges associated with widespread control over larger land areas and the range of objectives applied, together with novel approaches to reducing costs and improving success.

## 2 METHODS

We searched the scientific literature and consulted with experts to identify programmes that have attempted the eradication or complete removal of mammalian IAS from large contiguous land masses in Northern Europe. We did not consider projects based on small (<10 km^2^) land areas, typically islands, as these have been documented elsewhere.^9^, ^19^ We identified 15 documented large‐scale eradication or removal programmes of mammalian IAS from Britain, Ireland and Belgium. These related to seven species, including edible dormouse (*Glis glis*, L.), muskrat (*Ondatra zibethicus*, L.), coypu (*Myocaster coypus*, Molina), Himalayan porcupine *(Hystrix brachyura*, L.), American mink (*Neovison vison*, Schreber), grey squirrel (*Sciurus carolinensis*, Gmelin) and Pallas' squirrel (*Callosciurus erythraeus*, Pallas). In addition, the programme to remove the resident breeding ruddy duck (*Oxyura jamaicensis*, Gmelin) population from the United Kingdom^25^ was included for comparative purposes.

In Great Britain there were also a number of historic schemes for the local control of rabbits (*Oryctolagus cuniculus*, L. 1758), grey squirrels, mink and coypu, based on government support for local control groups or provision of bounties.^26^, ^27^, ^28^ In other parts of Europe, species such as muntjac (*Muntiacus reevesi*, Ogilby), muskrat and racoon (*Procyon lotor*, L.) are subject to ongoing control, but without eradication as the aim.^20^, ^29^, ^30^, ^31^, ^32^ These have the objective of reducing local damage and spread and do not constitute eradication or total removal. They were not considered when calculating the success of attempted eradication or removal programmes. Where possible, we extracted details of the objectives, timing, area, effort and outcome of each programme.^28^, ^33^, ^34^, ^35^, ^36^, ^37^, ^38^, ^39^, ^40^, ^41^, ^42^, ^43^, ^44^, ^45^, ^46^, ^47^, ^48^, ^49^ The area of control was calculated as the maximum polygon drawn around the outermost locations where animals were reported or captured, constrained by coastlines to avoid the inclusion of sea. Success was defined as reported confidence that the animals had been removed from the main area targeted by the programme. For some programmes, such as the removal of mink from the Scottish Highlands,^42^, ^46^ mink in the Uists^48^ or grey squirrels from Anglesey^44^ or Thetford,^41^ extant populations continued to exist adjacent to the areas targeted for clearance, and buffer zones were in place where control continued in an attempt to maintain targeted cleared areas until such time as control operations could be extended. In these cases, we only used data related to the cleared area, not the neighbouring buffer zone.

The effort involved in each programme was presented as the number of man‐years of full‐time trapper effort, which was the most common unit of effort presented across the studies, particularly for the older programmes. This could not be obtained for the edible dormouse control in the early 1900s^33^ or highland mink control work, which relied on volunteer effort.^42^, ^46^ In some cases, further details of equipment and logistic costs, management input or associated research were also given, but understanding these costs was complicated by the studies covering 11 different decades. To convert effort to an estimate of total cost, we considered a range of costs per trapper‐year of effort, to reflect the added costs of equipment, travel and oversight which were not consistently reported in the literature. A range of three figures were used to encompass this uncertainty ($US 50 k, 100 k or 200 k per trapper‐year) and this range of estimates is presented in the graphs when comparing with other costed eradications from the literature.

## 3 RESULTS

The objectives of the different programmes fell into three categories.

*Eradication* – the complete removal from an area, with no immediate prospect of recolonisation from neighbouring areas (edible dormouse,^33^ coypu,^39^ porcupine,^40^ mink on Harris,^43^ musk rats,^34^, ^35^ Pallas' squirrel^45^).

*Complete removal* from an area but with ongoing effort to maintain the area as clear. This may include the use of a buffer zone or fences to prevent recolonisation from extant populations in neighbouring areas (grey squirrels on Anglesey,^47^ mink on the Uists,^48^ mink in the Scottish Highlands^42^, ^46^) and/or continued monitoring and control within the cleared area to prevent the re‐establishment of colonising individuals (UK ruddy duck^25^).

*Control* within an area to reduce abundance, associated damage and the risk of spread, where complete removal would be desirable but is not an explicit objective (early grey squirrel, copyu, mink and rabbit programmes,^26^, ^27^ most traditional pest control, gamekeeping and wildlife damage management).

The details of the 15 large‐scale eradication/removal programmes are presented in Table 1; of these, 80% (*n* = 12) were considered successful. Data on worldwide island eradications were used for comparative purposes. Details are available of the costs of 41 eradications of a range of mammal species on islands of varying sizes,^19^ and in relation to 285 successful eradications of rodents on islands, and a further 53 unsuccessful examples.^20^ The larger‐scale programmes described here were some orders of magnitude larger than either set of small island cases, which provided the vast majority of reported eradications (Table 2).

**Table 1:** Data on large‐scale mammal eradications in Britain, Ireland and Belgium, obtained from the literature

Species	Years	Region	Area (km^2^)	Trapper‐years	Animals removed	Success	References
Edible dormouse	Early 1900s	Bedfordshire, England	?	?	?	No	33
Muskrat	1932–1935	Shropshire, England	1813^a^	61	3052	Yes	29, 30, 34, 35
Muskrat	1932–1937	Scotland	2815^a^	35.5	1248	Yes	29, 30, 34, 35
Muskrat	1933–1935	Surrey, England	96^a^	8^b^	169	Yes	29, 30, 34, 35
Muskrat	1932–1935	Sussex, England	81^a^	18^b^	52	Yes	29, 30, 34, 35
Muskrat	1933–1935	Clare/Tipperary, Ireland	414^a^	21	487	Yes	37
American mink	1964–1969	Great Britain	184 000^a^	77	ca 5000	No	28, 36
Himalayan porcupine	?–1979^c^	Devon, England	280	9	6	Yes	62
Coypu	1981–1989	East Anglia, England	19 210^a^	192	34 822	Yes	39
Grey squirrel	1998–2001	Thetford, England	17–46^d^	1.6^d^	2209	No	41
American mink	2001–2005	Hebrides – Uists, Scotland	850	23.5	228	Yes	48
American mink		Highland, Scotland	29 000				42, 46
Grey squirrel	1998–2013	Anglesey, Wales	710	30	6397	Yes	44, 47
American mink	2007–2013	Harris and Lewis, Scotland	2611	78	1514	Yes	43
Pallas' squirrel	2005–2011	Belgium	2.7^a^	2.3	248	Yes	46

a Area based on a maximum convex polygon drawn around the outmost reported sightings/captures from maps in the literature, or in the case of the 1960s mink programme, from records in the UK National Biodiversity Network up to 1970, excluding areas of open sea. For Pallas' Squirrel this is larger than the area of captures in the literature^46^ as it also includes sightings.

b Estimates from correspondence in the UK national records archive.

c Start date unclear from the literature.

d This includes area and trapper effort throughout the core and buffer zones.

**Table 2:** The area of reported successful invasive mammal eradications, based on two published reviews^16^, ^17^ in comparison with this review

	Rodents on islands^20^	Mammals on islands^19^	This study
*N*	285	41	11
Mean (km^2^)	1.67	11.70	2627.63
SE	0.46	4.46	1841.31
Min	0.01	0.01	2.70
Max	113	122.5	19 210.0

**Figure 1:**
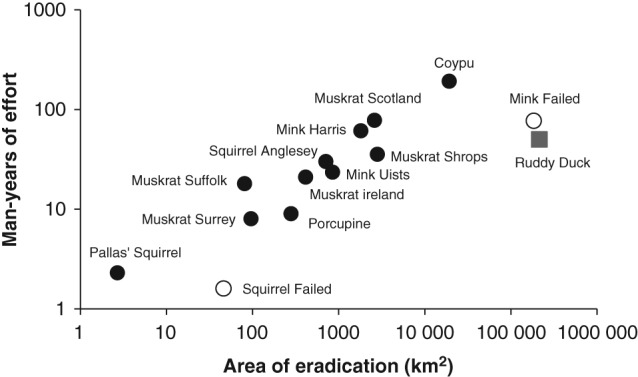
The relationship between the area and the manpower required to achieve eradication for 11 large‐scale invasive alien mammal eradications in Great Britain and Ireland (closed symbols). Also included for comparison are the failed mink eradication from the 1960s, a failed squirrel campaign from Thetford and the removal of a bird – the ruddy duck.^25^

The manpower estimates (log) for the 11 successful large‐scale programmes for which data on effort were available were entered into a model including area (log), number of animals removed (log) and year completed. This identified a significant relationship between the area over which removal took place and the reported manpower to achieve this (*n* = 11, *F* = 73.8, *P* < 0.001, *r* ^2^ = 0.879) (Fig. 1). Neither the addition of the number of animals removed (*P* = 0.102) nor the addition of the year of completion (*P* = 0.833) had any significant effect on the relationship.

**Figure 2:**
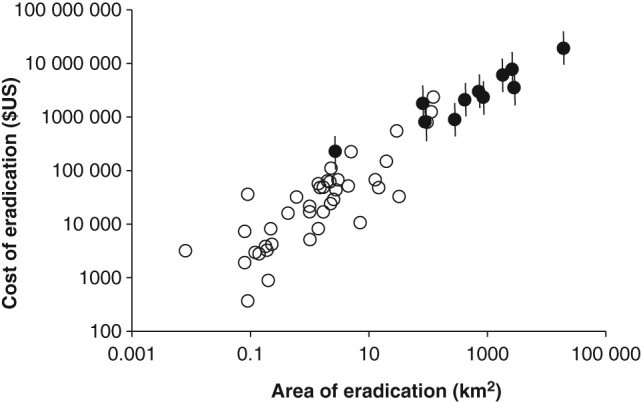
The relationship between the area of invasive mammal eradications and their cost. The open circles relate to island mammal eradications worldwide.^19^ The closed circles are the 11 large‐scale invasive alien mammal eradications in Great Britain, Ireland and Belgium, with the range of cost estimates represented by the vertical bars.

Combining the estimated costs of these large eradications (using the median estimate of $US 100 k per man‐year of effort) with the reported costs of small island eradications from the literature^19^ in a regression model (Fig. 2) found a significant relationship between cost and area (*n* = 51, *F* = 300.6, *P* < 0.001, *r* ^2^ = 0.857). The eradication programmes from larger land masses were significantly larger, but there was no evidence that the costs of island or large‐scale programmes differed in their relationship with area (*P* = 0.201).

**Figure 3:**
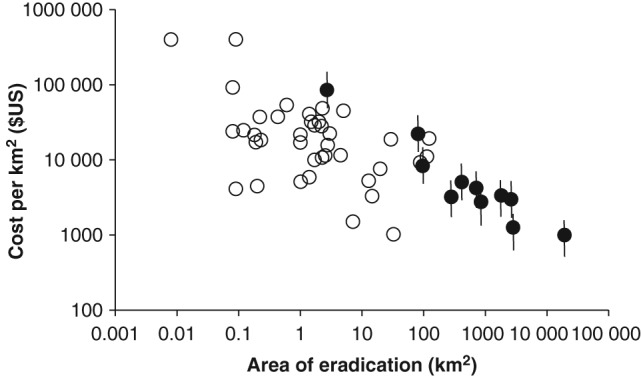
The relationship between the area of invasive alien mammal eradications and the cost per unit area (n = 51, F = 41.9, P < 0.001, r ^2^ = 0.456). The open circles relate to island mammal eradications worldwide.^19^ The closed circles are the 11 large‐scale invasive alien mammal eradications in Great Britain, Ireland and Belgium, with the range of cost estimates represented by the vertical bars.

Applying a similar model to the estimated cost per unit area of larger eradications and small island eradications from the literature found a significant negative relationship between cost per unit area and area of eradication (*n* = 51, *F* = 41.9, *P* < 0.001, *r* ^2^ = 0.456). There was no evidence that the cost per unit area of island or larger‐scale programmes differed in their relationship with area (*P* = 0.201) (Fig. 3).

## 4 DISCUSSION

The total number of large‐scale mammalian IAS programmes is small, and certainly considerably fewer than have taken place on islands (<10 km^2^). The examples described here comprise a significant proportion of such large control programmes, for Europe at least, and these have been orders of magnitude larger than those on islands worldwide. Nevertheless, the effort required to achieve eradication in these large programmes appeared to follow a similar form to that observed on islands.

The large‐scale mammal eradications or removals for which effort data were available demonstrated a consistent relationship between area and the effort required to achieve eradication. This is unsurprising, as all were based on similar methodologies, the main control method involving the daily checking of static live‐capture or kill traps by professional staff. The effort associated with the use of volunteers by some programmes was not directly comparable, but is likely to have been significantly cheaper.

The costs and effectiveness of island eradications are well described,^19^, ^20^ with the most important factors influencing cost being island area, remoteness and date, with more recent eradications becoming cheaper as experience has been gained. From this study, the costs of eradication were linked primarily to the size of the area involved, following the same relationship as observed on islands. However, programmes on larger land masses introduce uncertainties not observed on islands. For island control, estimating the area to be covered before work commences is straightforward, but on larger land masses it is determined by the spread of the population, which may be unknown before work commences and can change. This adds considerable uncertainty to initial estimates of cost, with consequences for the planning and procurement of such programmes. However, cost of removal per unit area was found to decrease as the area involved increased. Crudely, a doubling of the area controlled resulted in a 10% reduction in cost per unit area. This has important implications when planning large programmes, for example mammalian IAS removal at a national level, as proposed by Predator‐free New Zealand.^24^ We found that the cost of eradication, using manpower as a proxy, was most strongly influenced by the extent of the area over which eradication occurred, while additional information on the number of animals removed did not significantly improve the model. When planning the resources required for an eradication campaign, it is rare that the number of animals is known with confidence, and obtaining this information can lead to significant extra costs and delays. By contrast, the expected area to be covered can be assessed more directly, and appears to provide a better basis for prediction. However, this assumes that the same relationship holds for different species or groups. By comparison, the effort required to remove the ruddy duck by shooting^25^, ^49^ was significantly less than would have been expected for a mammal. This highlights the caution needed if extrapolating these findings to different control methods or species. The failed mink eradication of the 1960s was based on less effort than would have been expected from other programmes, and it is tempting to blame its failure on insufficient effort given the rapid spread of this species from multiple foci, including extant mink farms, with the continued risk of further escapes. Similarly, the unsuccessful Thetford squirrel removal used manpower at the low end of what would be expected given the results of other successful eradications.^41^

Studies describe an 84% success rate of island eradications, based on 338 cases.^20^ Of the 15 programmes on larger land masses described here, the success rate was 80%. A review of 136 eradication campaigns against 75 species of invasive alien invertebrates, plants and plant pathogens tested which factors were significantly related to eradication success.^17^ In these cases, only the spatial extent of the programme was significantly related to the eradication outcome: local campaigns were more successful than regional or national campaigns. This paper does not provide evidence to support a similar conclusion for mammalian IAS, suggesting that these larger‐scale programmes were no less successful than those conducted on islands. However, care is needed in defining success, as in three cases (Scottish Highland mink, Uist mink and Anglesey squirrels) this constituted complete removal rather than true eradication, and the risk of reinvasion remained. The figures on success are also only for those cases where eradication or complete removal was attempted. Examples of local control to reduce conflicts or spread were not included. The three removal programmes fall between the commonly used definitions of eradication or ongoing control^22^ and are worth considering in more detail.

The Scottish Mink Initiative, and its predecessor the Cairngorms Water Vole Conservation Project,^42^, ^46^ initially removed mink from 6000 km^2^ centred on the Cairngorms National Park, but instead of holding an arbitrary line, expanded control into adjacent source habitat from where reinvading mink originated.^50^, ^51^ It now enacts mink control by volunteers supported by 3–4 staff over 29 000 km^2^ bounded by the North Sea, the Cairngorms mountains and a semi‐permeable barrier to mink and buffer zones. Its objective is ‘to keep the area free of established adult female mink in spring’. This approach has been adopted by a range of other, local mink control programmes,^52^, ^53^ although there is currently no prospect of the national eradication of this species from Great Britain.

The mink programmes on the Uists and Harris provide a sequence with escalating objectives, with the limited objective of removing mink to a buffer zone for the Uist work,^48^ while the subsequent Harris project aimed to achieve eradication throughout the archipelago.^43^ The Harris work is also awaiting confirmation of final success, which may add to the time and effort estimates.

On Anglesey, grey squirrel control eradicated this species from the island itself, but ongoing control in a 2 km wide and 5 km long adjacent coastal mainland area was necessary to prevent individuals from crossing the narrow sea‐strait via road or rail bridges and recolonising Anglesey.^44^, ^47^ The existence of continuous forest habitat running deeper inland means that this arbitrary buffer zone is rapidly recolonised owing to the proximity of uncontrolled populations.^54^ This scenario has led to proposals for squirrel eradication over a wider 90 km^2^ mainland area. This landscape is advantageous as it is bordered on three sides by treeless mountain ranges and coast. Should eradication be successful here, ongoing control across a 6 km wide area of wooded landscape would be required to prevent recolonisation, but it would greatly reduce the probability of island reinvasion. Elsewhere in Great Britain, local grey squirrel control programmes, often conducted by volunteers, can reduce local abundance but have not altered distribution. It has been argued^55^ that regional coordination of isolated local grey squirrel control efforts can facilitate native red squirrel (*Sciurus vulgaris*, L.) persistence, although this requires continued resource input.

The resource challenges posed by grey squirrel control are reflected in a useful example of an experimental programme that failed to achieve landscape clearance but quantified control effort and returns. This 3 year control programme was conducted in Thetford forest, East Anglia, a managed coniferous plantation that contained a nationally important remnant red squirrel population vulnerable to competitive replacement by the congener.^41^ Control was carried out over a maximum area of 46 km^2^ to reduce grey squirrel abundance to low levels and ideally to zero within a central 17 km^2^ forest area, using approximately 0.5 man‐years of effort per year. However, the number of grey squirrels in the core area continued to increase through time, and thus the level of control was not sufficient to prevent grey squirrel occupation.^41^ The study included monitored radio‐tagged squirrels present during the control period, which remained uncaught, demonstrating that presence was in part due to the failure to remove resident animals.

There are additional examples of the successful use of such intermediate strategies, removing mammalian IAS to a barrier or maintaining a buffer zone to prevent recolonisation. These include the use of fenced ‘mainland islands’ in New Zealand,^56^, ^57^ mongoose (*Herpestes javanicus*, St‐Hiliare) control on Okinawa^58^ and removal of feral swine (*Sus scrofa*, L.) from fenced areas in US national parks.^59^, ^60^ While requiring ongoing effort to maintain the buffer zone or fence, these provide many of the benefits of true eradication but with ongoing costs, as the buffer must be maintained forever or the invasion process will repeat itself. This incremental approach has been advocated as a unifying strategy for current regional control programmes, with the potential for eradication at some point in the future, for example through initiatives such as Predator‐free New Zealand.^24^

Ongoing control within an area to reduce abundance, associated damage and the risk of spread is widely undertaken by local interests, for example predator removal by gamekeepers, deer management and local pest control. It does not specifically include complete local removal or eradication as an objective, and effort is often determined by local perceptions of damage or financial incentives for the activity. In Great Britain, government funds were used to support such local activities between 1950 and 1970 in relation to coypu, mink, rabbit and grey squirrel control, providing funds for local control groups or bounties.^26^, ^27^ These may have reduced local abundance, but required continual investment. The use of bounties can be particularly problematic, as they provide an incentive to maintain the species in the area to ensure continued income.^61^ In no case did they lead to eradication, although they provided useful information to inform later eradication schemes, for example the coypu.^62^

While coordinated databases of island eradications are maintained,^21^ no such inventory of larger‐scale programmes exists. In addition to the programmes described here, studies describe the removal of an American beaver (*Castor canadensis*, Kohl) population from France^63^ and the continuing control of this species in eastern France, Belgium and Luxembourg.^64^, ^65^ In the Scottish Hebrides, work to remove introduced populations of hedgehog (*Erinaceus europaeus* L.) is ongoing,^62^ as are efforts to remove the monk parakeet (*Myiopsitta monachus*, Boddaert) from southern England.^66^

In spite of these large‐scale programmes spanning 11 decades, there was no suggestion that efficiency of programmes based on the employment of trappers improved over that period, and the basic approach of daily checking of static live‐capture or lethal traps remained unchanged. The majority of the cost of such programmes comprises the manpower costs of the trappers. More recently, the Scottish Mink Initiative and regional grey squirrel control have relied on public volunteers to undertake much of the trapping. This approach greatly reduces the manpower costs compared with dedicated teams of trappers and has been successful in mammalian IAS removal over large areas, but current examples require continued inputs at their borders and to remove dispersing animals. The use of this voluntary approach to achieve permanent eradication has yet to be demonstrated but has clear potential for some species and habitats. New technologies also offer the prospect of more labour‐efficient approaches. Examples include self‐reporting or self‐resetting traps,^67^ the use of camera^68^ or footprint traps^69^ to record presence without the need for daily checking, the optimisation of trap use^70^ and the use of contraception in place of, or as an adjunct to, lethal control in specific circumstances.^71^

Lastly, these eradications have proven to be considerably geographically larger than is the case for islands, with greatly increased total costs as a consequence, although mitigated to some extent by the economies of scale described here. Although accepted best practice is for there to be a rapid response to remove new invasive species,^17^ only the removal of Pallas' squirrel, muskrat and porcupine conceivably fits this description from the cases reviewed here, along with the failed edible dormouse eradication, although few details of this are available. The other cases, including the grey squirrel, were large programmes to remove species that had been in place for considerable periods of time and spread widely, with consequently large costs. We note that early intervention was a key factor in the successful eradication of grey squirrels from Adelaide^72^ and the subsequent extinction of this species from Australia. Our study has demonstrated the high costs associated with a failure to respond quickly, and this should act as an incentive to adopt rapid reaction as best practice, both to minimise environmental impacts and financial costs and to reduce the number of animals whose welfare is affected by the need for control.
